# Endurance Sports and Atrial Fibrillation: A Puzzling Conundrum

**DOI:** 10.3390/jcm13247691

**Published:** 2024-12-17

**Authors:** Marina Ostojic, Mladen Ostojic, Olga Petrovic, Olga Nedeljkovic-Arsenovic, Francesco Perone, Marko Banovic, Tamara Stojmenovic, Dragutin Stojmenovic, Vojislav Giga, Branko Beleslin, Ivana Nedeljkovic

**Affiliations:** 1 Cardiology Clinic, University Clinical Center of Serbia, 11000 Belgrade, Serbia; ostojic.mladen@yahoo.com (M.O.); opetrovic1976@gmail.com (O.P.); markobanovic71@gmail.com (M.B.); voja2011@yahoo.com (V.G.); branko.beleslin@gmail.com (B.B.); ivannanedeljkovic@yahoo.com (I.N.); 2 School of Medicine, University of Belgrade, 11000 Belgrade, Serbia; olganedeljkovic@gmail.com; 3 Radiology and MRI Department, University Clinical Center of Serbia, 11000 Belgrade, Serbia; 4 Cardiac Rehabilitation Unit, Rehabilitation Clinic “Villa delleMagnolie”, 81020 Castel Morrone, Italy; francescoperone1988@gmail.com; 5 Faculty of Physical Culture and Sports Management, Singidunum University, 11000 Belgrade, Serbia; antictamara@hotmail.com; 6 Department of Physiology, Faculty of Medical Sciences, University of Kragujevac, 34000 Kragujevac, Serbia; dragutin.stojmenovic@gmail.com

**Keywords:** atrial fibrillation, endurance sport, endurance activity, cardiovascular prevention

## Abstract

The confirmed benefits of regular moderate exercise on cardiovascular health have positioned athletes as an illustration of well-being. However, concerns have arisen regarding the potential predisposition to arrhythmias in individuals engaged in prolonged strenuous exercise. Atrial fibrillation (AF), the most common heart arrhythmia, is typically associated with age-related risks but has been documented in otherwise healthy young and middle-aged endurance athletes. The mechanism responsible for AF involves atrial remodeling, fibrosis, inflammation, and alterations in autonomic tone, all of which intersect with the demands of endurance sports, cumulative training hours, and competitive participation. This unique lifestyle requires a tailored therapeutic approach, often favoring radiofrequency ablation as the preferred treatment. As the number of professional and non-professional athletes engaging in high-level daily sports activities rises, awareness of AF within this demographic becomes imperative. This review delivers the etiology, pathophysiology, and therapeutic considerations surrounding AF in endurance sports.

## 1. Introduction

Atrial fibrillation (AF), the most prevalent sustained arrhythmia in the general population, stands as a leading contributor to global morbidity and mortality [1]. Its well-documented associations with an elevated risk of stroke, dementia, heart failure, and increased cardiac and overall mortality accentuate its impact on global health [1]. In recent years, the prevalence of AF has been steadily increasing, with expected projections to double by 2060 [2]. Past guidelines for managing AF state that moderate regular physical activity is recommended to prevent AF. However, firstly, there were small, then extensive epidemiological studies and guidelines that recognized and confirmed the association between AF and exercise, particularly persistent endurance [1,3,4,5]. This knowledge carries significant implications for treating and monitoring a rapidly expanding population of endurance athletes (EAs). However, the existing data on the association between AF and endurance exercise (EE) remain inconclusive, giving rise to numerous controversies and unanswered questions.

EAs, such as long-distance runners, cyclists, and swimmers, are at higher risk for developing AF due to the intense, prolonged cardiovascular demands of their training [3,4,5]. EE leads to adaptive changes in the heart, including atrial enlargement and alterations in electrical conduction, which can improve efficiency during exercise but also create conditions that promote AF. The shift in autonomic tone, with increased parasympathetic (vagal) activity and reduced sympathetic drive, combined with elevated inflammation and oxidative stress from excessive training and insufficient recovery, may contribute to atrial fibrosis, a key risk factor for AF [6]. Also, genetic predisposition in athletes associated with structural heart disease can trigger the onset of AF [7]. In this context, we provide a comprehensive review of the current understanding of the primary mechanisms of AF, along with diagnostic and therapeutic strategies within this population.

## 2. Epidemiology

The general population displays varying prevalence rates of AF (paroxysmal or persistent), ranging from 0.5% in the 45–54 age group to 4% in the 65–74 age range [5]. Studies and case controls from the late 1990s highlighted a heightened AF risk associated with prolonged, vigorous training [8], a trend later confirmed by subsequent research and meta-analyses [9,10,11]. Presently, endurance sport is a well-acknowledged and accepted cause of AF [1,5,6], with a 2 to 10 times higher frequency in high-intensity EA in contrast to sedentary counterparts (Table 1) [1,5,6,9].

The Physicians’ Health Study prospectively analyzed 16,921 healthy men, the types and intensity of physical exercise, and the associated risk of AF. Vigorous exercise was correlated with an increased risk of AF in individuals below 50 years old and among joggers [12]. Compared with non-athletes, men who jogged five to seven times per week had an increased risk of developing AF (risk ratio [RR]: 1.53, 95% confidence interval [CI]: 1.12 to 2.09; *p* < 0.01).

A study by Andersen et al. of over 52,000 cross-country skiers in Sweden demonstrated an evident association between completed 90-km races and AF incidence. AF appeared in 681 skiers (hazard ratio [HR]: 13.2; 95% CI: 12.3 to 14.3/10,000 person-years at risk) with the expanded proportion of arrhythmia in skiers who completed more 90-km races (HR: 1.29; 95% CI: 1.04 to 1.61 for >5 completed races vs. 1 completed race) [13].

Accordingly, the threshold theory suggests that over 1500 to 2000 lifetime training hours may heighten the risk of AF [8,14]. A “J-shaped” pattern in the exercise-AF relationship has also been identified [8,14], with routine mild to moderate exercise protecting against cardiovascular disease and AF. In contrast, prolonged EE may increase AF burden [3,5,6,12,13], particularly in men. Surprisingly, this association is not consistently observed in women, explained by a gender paradox characterized by lower sympathetic tone, more balanced autonomic function, and lower blood pressure [8].

Recent Drca N et al.’s matched cohort study involving Swedish female athletes revealed a heightened risk of AF among female EA compared to controls (HR 3.67), thereby challenging pre-existing assumptions [15]. This discovery holds particular significance as it conflicts with the trends observed in numerous prior studies, where small sample sizes and inadequate exposure to prolonged, intense physical activity often limited the representation of women.

Today, in everyday practice, exercise-induced AF is mainly seen in male athletes of middle age and tall stature who have been participating in long-term (>10 years or exceeding 1500–2000 lifetime training hours) endurance sports (marathon, cross-country skiing, cycling) [8,14].

**Table 1:** Studies that focus on the prevalence and risk of atrial fibrillation in various endurance athletic populations.

Authors	Year	Male (%)	Study Design	Sport	Age (Mean ± SD, Year)	RR (95% CI) for Athletes	AF/ Athletes (N)	Outcomes
Karjalainen et al. [16]	1998	100	Case-control	Runners	47 ± 5	5.5 (1.3–24.4)	12/228	Prevalence of AF
Heidbuchel et al. [17]	2006	83	Cohort	Mixed sports	53 ± 9	-	25/31	Prevalence of AF
Baldesberger et al. [18]	2008	100	Case-control	Former elite cyclists	67 ± 7	14.4 (0.8–261.1)	6/62	Prevalence of AF
Molina et al. [3]	2008	100	Cohort	Marathon runners	39 ± 9	8.8 (81.3–61.4)	9/183	Prevalence of AF
Pelliccia et al. [19]	2005	71	Cohort	Mixed sports	24 ± 6	-	5/177	Prevalence of AF
Wilhelm et al. [20]	2011	50	Cohort	Non elite runners	42 ± 7	-	4/122	Prevalence of AF
Grimsmo et al. [21]	2010	100	Cohort	Cross-country ski racing	54–62 years—Group I 72–80 years—Group II 87–92 years—Group III	-	13/78	Prevalence of AF

SD—standard deviation, RR—relative risk, AF—atrial fibrillation.

## 3. Etiology and Pathophysiology

**Figure 1:**
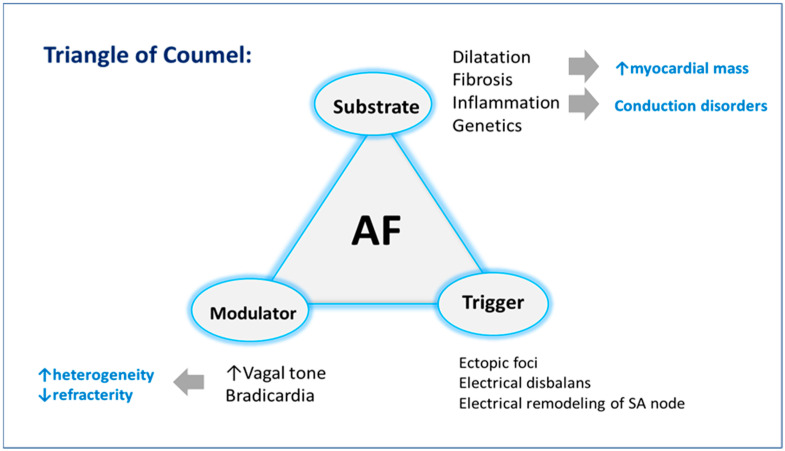
Mechanism of atrial fibrillation in endurance athletes. AF—atrial fibrillation.

Several knowledge gaps exist regarding the pathophysiological mechanisms conducting the progression of AF in athletes. The evidence indicates a multifactorial influence involving electrical remodeling, atrial enlargement, atrial fibrosis, autonomic tone alterations, and chronic inflammation development (Figure 1).

### 3.1. Remodeling of Left Atria

The diameter of the left atrium (LA) is an independent predictor of AF in healthy middle-aged individuals [22]. In a population of EA, primarily marathon runners, an enlarged LA volume was associated with a higher risk of AF [3]. These findings refer to middle-aged EAs with over 1500 training hours [14]. Meanwhile, Pelliccia et al. observed that of 1777 young competitive athletes, 347 exhibited LA enlargement (40 mm) without a greater risk of AF [19]. The indicated enlargement was explained as physiologic adaptation in this age group of athletes [23]. Repeated pressure and volume overload during long exercise sessions might lead to LA enlargement and microtrauma, inflammation, and fibrosis, which are all potential substrates for arrhythmias [24]. The data are conflicting regarding female competitive athletes, as endurance activity does not negatively affect their LA function after four months of training [25]. Also, female veteran EAs have less pronounced atrial remodeling [26].

#### 3.1.1. LA Enlargement and Left Ventricular Hypertrophy

Left ventricular hypertrophy (LVH) is characterized by the thickening of the left ventricular walls, often from chronic pressure and volume overload, or both, most commonly seen due to hypertension, aortic stenosis, or hypertrophic cardiomyopathy [27]. Increased left ventricular end-diastolic pressure, impaired diastolic function, and neurohormonal activation led to elevated pressure and enlargement of the LA contributing to arrhythmias such as AF [28]. In athletes, cardiac remodeling from intense training can mimic LVH. Athletes’ LVH is symmetrical, reversible, and, most importantly, associated with preserved diastolic function [29]. Endurance sports induce volume overload, leading to proportional LVH, whereas resistance sports generate pressure overload and may lead to concentric LVH. Nevertheless, prolonged training with high cardiac output may lead to the enlargement of LA, increasing the risk for AF in this population. The key finding in endurance athletes presents a distinction between whether atrial enlargement is a healthy adaptation or a sign of underlying disease [30]. Echocardiographic assessment can show us good remodeling of atria when normal reservoir function and myocardial flexibility are present [30].

#### 3.1.2. LA Fibrosis

Fibrosis in the LA may play a crucial role in developing supraventricular arrhythmias [31]. The current understanding of fibrosis linked to exercise-induced AF is mainly derived from animal studies. The formation and advancement of atrial fibrosis are fundamental to structural remodeling in AF and create a substrate for recurrent AF [31]. Two studies involving exercise-exposed vs. sedentary Wistar rats [32,33] support these findings. After sixteen weeks of exercise, fibrosis and biomarker levels increased markedly in the atria and ventricles of Wistar rats compared to controls. This included elevated levels of fibronectin 1, TGF β1, matrix metalloproteinase 2, tissue inhibitor of metalloproteinase 1, and procollagen I and III. Moreover, stopping exercise reversed the fibrosis changes induced by exercise over eight weeks [33]. In contrast, Guasch et al. found that while a 16-week exercise intervention led to atrial fibrosis in rats, this fibrosis persisted even after detraining [34].

Wilson et al. observed myocardial fibrosis in six of twelve veteran male EAs, as detected by late gadolinium enhancement (LGE) on cardiac magnetic resonance (CMR). No LGE was found in sedentary controls. The years in training and participation in competitive marathons were identified as independent predictors of LGE on CMR [35]. Peritz et al. assessed LA fibrosis using LGE magnetic resonance imaging in 20 Masters athletes compared to healthy non-athletes. They found that LA volumes were significantly larger in athletes (74.2 mL ± 14.4) compared to controls (60.8 mL ± 21.4) (*p* = 0.02). Furthermore, the mean LA fibrosis score was 15.5% ± 5.9 in athletes versus 9.6% ± 4.9 in the healthy control group (*p* = 0.002) [36].

Repetitive cardiovascular strain from endurance training causes the heart to adapt by enlarging the atria, which stretches the atrial tissue and promotes fibrotic changes [30]. This fibrosis disrupts the normal conduction of electrical signals, creating areas of electrical irregularity that foster re-entry circuits and chaotic activity, key features of atrial fibrillation (AF). In athletes who overtrain without adequate recovery, the balance between beneficial cardiac remodeling and pathological fibrosis can shift, leading to excessive scar tissue formation and risk of AF. Insufficient rest impairs the body’s ability to repair and manage these structural changes, further raising the likelihood of arrhythmias [37].

#### 3.1.3. Cardiac Inflammation and Biomarkers

The role of inflammation in endurance-related AF remains controversial. However, along with oxidative and metabolic stress, cardiac inflammation may play a key role in electro-autonomic remodeling and tissue alterations. Additionally, migrating inflammatory cells and pro-inflammatory cytokines may create a favorable environment for initiating and maintaining AF. Several studies have linked AF with elevated levels of C-reactive protein (CRP) [38], and further research has identified tumor necrosis factor-alpha (TNFα), interleukin-6, and interleukin beta-1 as significant inflammatory markers. Animal studies [32] have shown that exercise-induced atrial remodeling and inflammation in trained mice can be prevented by treatment with the TNFα inhibitor Etanercept. Moreover, these changes were absent in mice lacking the TNFα gene.

In recent years, significant progress has been made in identifying potential biomarkers of AF. Micro-RNAs (miRNAs), non-coding genome regions, regulate ion channel remodeling during arrhythmias by modulating gene expression in cardiomyocytes. Several miRNAs, including miR-1, miR-26a, miR-29b, miR-30a, and miR-133a, have emerged as important mediators of pro-arrhythmogenic remodeling [39]. Clauss et al. investigated blood levels of miRNAs after a marathon in athletes of varying performance levels [40]. Trained (‘elite’) runners exhibited more significant changes in several miRNAs compared to baseline values than less trained (‘nonelite’) runners. Specifically, levels of miR-1 and miR-133a significantly increased after the race in elite runners, while miR-26a decreased only in this group. The authors suggested that differences in miRNA expression levels may partly explain the fluctuating impacts of ‘potentially harmful strenuous exercise’ versus ‘beneficial moderate exercise’ in athletes after long-term EE [39].

### 3.2. Modulators of AF

#### Autonomic Activation

There are two forms of AF seen in athletes: predominantly vagal-mediated and predominantly adrenergically-mediated. Vagal AF, the common one, typically occurs at night when the vagal tone is heightened. Increased vagal tone can trigger AF by creating a macro-reentry pathway through the dispersion of the atrial refractory period [41]. Studies have also reported lower resting heart rates as a predictor of AF in long-term EA. In this population, high vagal tone is often associated with prolonged PQ intervals and first-degree atrioventricular block [42]. Additionally, higher vagal tone has been observed in non-athletes with a higher lifetime training duration (>4500 h vs. >1500 h, *p* = 0.002) [20].

Catecholaminergic bursts and high sympathetic activity during acute strenuous EE, such as high-intensity training or competition, can also lead to AF [20]. Adrenergically mediated AF is more commonly seen in young, healthy, and particularly overtrained athletes [37,43], as well as in older athletes with known heart conditions.

When compared, EAs are more likely to experience vagally-induced AF than adrenergically-induced paroxysmal AF (30 vs. 7 athletes, *p* < 0.05) [44]. Currently, many researchers are questioning the definition and diagnostic criteria for vagal AF, as this condition is still underrecognized and likely underdiagnosed in the growing population of EA.

### 3.3. Triggers

The main trigger for the onset of an AF episode is pulmonary vein ectopy. However, this is only slightly more common in EA, and further research is needed to clarify its connection with SEE. During intense exercise, athletes may experience dynamic fluid shifts, electrolyte imbalances, pH alterations, and dehydration, all of which can contribute to the onset of AF. Additionally, regular exercise may lead to gastroesophageal reflux, associated with the induction of AF [45].

The use of performance-enhancing drugs by both professional and amateur athletes is a well-known issue, but the link between these drugs and AF in EA remains speculative. In a study by Baldesberger, 44 of 62 veteran cyclists admitted to using substances such as amphetamines or anabolic steroids [18]. However, sports with high doping rates, such as bodybuilding and wrestling, have not shown an increased incidence of AF [46]. Also, there are speculations about the role of environmental factors in exercise-induced AF, but further investigation is needed to establish any connections.

## 4. Diagnosis

Clinical AF is diagnosed by a 12-lead electrocardiogram or a single-lead tracing of ≥30 s showing heart rhythm with no detectable repeating P waves and irregular RR intervals (when atrioventricular conduction is not impaired) [5].

AF in otherwise healthy young, middle-aged, and veteran athletes requires further evaluation by a cardiologist. A comprehensive patient history, including details on the frequency and duration of arrhythmia episodes, the severity of symptoms, and their relationship to sports activities, is crucial [5,6,47]. EA with AF typically reports more severe symptoms compared to sedentary individuals but often maintains higher levels of activity than non-athletes with the same condition [48].

A thorough investigation of all potential contributing factors is essential. A detailed social history, including the use of caffeine, alcohol, illicit drugs, and performance-enhancing substances, is necessary. Subsequent evaluation should include a physical examination, a 12-lead electrocardiogram (ECG), laboratory analysis (including thyroid function tests), and additional diagnostic tests such as echocardiography (including strain echocardiography), treadmill testing (selectively performed to evaluate exercise capacity and check for ischemia), Holter ECG (screening and diagnosis, also assessing AF burden and ventricular rate control at rest and during exercise), prolonged ECG monitoring (for suspected but undocumented episodes), and CMR imaging [1,5,6,49]. During this evaluation, it is important to rule out any underlying medical conditions that could be associated with AF, such as hypertension, coronary artery disease, hyperthyroidism, pericarditis, Wolff-Parkinson-White syndrome, hypertrophic cardiomyopathy, Brugada syndrome, or long QT syndrome [49].

### 4.1. Early Detection of Atrial Fibrillation

Early detection of AF is important for initiating timely treatment and preventing complications linked to this arrhythmia. International AF guidelines encourage systematic and opportunistic screening [1,5,6]. Numerous digital tools have been proposed and are now available [50]. Mobile health (mHealth) devices provide an opportunity for quick screening and should be part of innovative care delivery [51]. Studies with screening AF programs showed asymptomatic arrhythmia frequently, but data supporting the benefit of further treatment are lacking. Also, single-lead ECG interpretations by cardiologists currently show higher accuracy than the AI algorithms embedded in commercial devices [51]. The meta-analysis of 28 studies validated smart devices for diagnosing AF, and 15 studies for screening AF included smartphones with photoplethysmography (PPG) sensors, smart bands, and external electrodes providing single-lead ECG showed different accuracy based on technology and screened population. Single-lead ECG displayed a sensitivity range of 66.7% to 98.5% and specificity between 99.0% and 99.4%, while PPG sensors showed a sensitivity of 85.0% to 100% and specificity from 93.5% to 99.0%. The incidence of newly diagnosed arrhythmias ranged from 0.12% in healthy individuals to 8% among hospitalized patients [52]. MHealth technology has many advantages, such as portability, instant access, and direct communication, enabling quicker transmission of patient data and self-reported symptoms to healthcare providers, potentially transforming clinical care in a cost-effective way.

Furthermore, today, there are modern fitness devices that help monitor exercise intensity using various protocols. Heart rate monitoring maintains real-time tracking within target zones, while heart rate variability determines recovery and readiness for high-intensity workouts [53,54]. VO_2_max estimation offers insights into functional cardiovascular capacity and metabolic equivalents, measuring energy consumption during activities [54]. Combining self-reported perceived exertion (RPE) with device metrics provides accuracy, while GPS and accelerometry track speed, distance, and elevation for outdoor activities. Additionally, training load calculations suggest recovery time, and lactate threshold estimation helps optimize endurance intensity [55]. Together, these methods support effective training and recovery strategies.

### 4.2. The Role of Different Imaging Modalities in the Evaluation of the Left Atrium

Transthoracic echocardiography (TTE) is one of the initial diagnostic imaging techniques used in athletes with AF. In addition to using M-mode to assess LA diameter, LA volumes should be estimated to quantify LA size. The modified biplane Simpson’s rule with planimetry is considered the most accurate and commonly used approach. The ellipsoid and biplane area–length methods, which use various LA diameters and areas to calculate LA volumes, are also utilized [56]. Real-time 3D echocardiography provides a more accurate assessment of LA volumes [57].

Furthermore, it is possible to assess LA function. While no single parameter perfectly defines LA function, measures such as transmitral peak A wave velocity [58], its velocity-time integral, and atrial fraction are recognized indicators of LA contractile function. However, these measurements are only possible in the presence of sinus rhythm. The LA function index (LAFI) allows the evaluation of the LA function even in AF [58]. Additionally, LA ejection fraction (LAEF) and LA expansion index (LAEI) have been used in both sinus rhythm and AF [59].

Recently, strain analysis has been employed to assess LA function. Atrial strain measures the deformation of the atrial myocardium, while strain rate examines the rate of change in strain throughout the cardiac cycle. This enables the evaluation of atrial reservoir function (during systole) and conduit and contractile function (during diastole) [59,60,61].

To date, CMR is the gold standard for quantifying LA size. Due to its three-dimensional capabilities and high spatial resolution, CMR provides an accurate assessment of the LA. The modified Simpson’s rule is applied for assessing LA areas using LA areas from consecutive cross-sectional images [62]. However, MRI has limitations, including time-consuming data acquisition and analysis. As a result, MRI is not commonly used in clinical practice to assess LA size.

Multi-slice computed tomography (MSCT) also allows for a three-dimensional assessment of LA size, offering high spatial and temporal resolution [63]. However, it is not routinely used due to the need for contrast and radiation exposure. Imaging modalities for the evaluation of the LA are shown in Table 2.

## 5. Management of AF in Endurance Athletes

**Figure 2:**
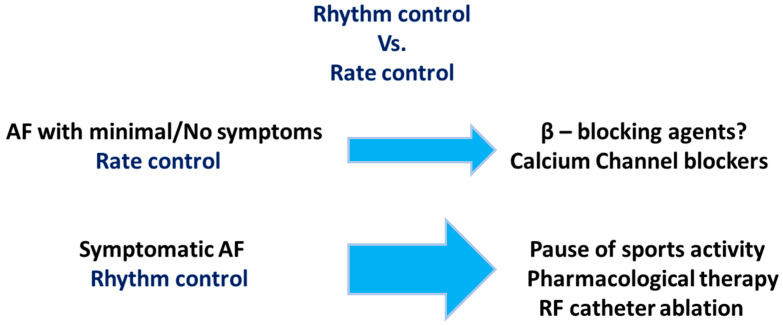
Treatment strategy for atrial fibrillation in endurance sports. AF—atrial fibrillation, RF—radiofrequency ablation.

A key aspect of managing AF in EA is respecting the athlete’s decisions while offering the most appropriate therapeutic options for each situation. The management approach may vary depending on the severity of symptoms and the context in which AF episodes occur (Figure 2).

### 5.1. Exercise Reduction

The initial clinical strategy for managing AF in EA is to reduce exercise volume. To stabilize sinus rhythm in the early stages of paroxysmal AF, athletes are advised to stop training for approximately two months [48]. Although these recommendations are primarily based on expert opinion and observational studies, reducing exercise loads can be beneficial in decreasing the substrates and triggers for AF in this group. Positive responses to the modification of sports activities have been reported in top-level athletes, with up to 30% experiencing fewer episodes of AF after reducing their athletic activities [44]. The latest guidelines suggest temporarily discontinuing intensive sports participation until the underlying cause of arrhythmia is identified and corrected [5,48].

### 5.2. Pharmacological Treatment

If the initial strategy of reducing exercise frequency, duration, or intensity is unsuccessful, further treatments should be considered (Figure 2). Management options for AF in EA, similar to those in the general population, include pharmacological (rate or rhythm control) and non-pharmacological approaches. A rate control strategy is preferred when arrhythmia episodes are accompanied by minimal or no symptoms [48,64]. However, this approach can be challenging for highly dedicated athletes, as it may impair performance during training and competition, with more symptoms than a sedentary population [65]. Also, rapid atrioventricular nodal conduction of AF during exercise may lead to significant symptoms (fatigue, dizziness, or syncope). Additionally, athletes typically have a naturally slow baseline sinus rhythm, and there is no clear definition of target resting or peak exercise heart rates under this therapy. Beta-blockers are poorly tolerated. Sotalol in this population can manifest side effects [66]. Small studies advocate that calcium channel blockers are superior in improving heart rate control and [67] affect exercise capacity to a lesser extent [67] in comparison to beta-blockers. The use of digoxin is not recommended due to its negative dromotropic effects. Caution is also advised when prescribing beta-blockers due to their potential as doping agents in certain sports. The 2020 ESC Guidelines on Sports Cardiology and Exercise in Patients with Cardiovascular Disease suggest examining asymptomatic individuals regarding adequate control within AF through an exercise stress test or ECG monitoring during training. All sports participation is possible in asymptomatic, well-controlled individuals. Also, careful titration and dosing of used drugs should be instituted (Class IIa, Level of Evidence C) [48].

For athletes with clinically significant symptoms during exercise or at rest, efforts should be made to maintain normal sinus rhythm, typically through a trial of an antiarrhythmic agent, which can be very complicated [68,69]. No prospective randomized trials assess the effectiveness and safety of antiarrhythmic agents in EA. Selecting an appropriate drug should be guided by the best available evidence from other patient populations with AF.

Class I and III antiarrhythmic drugs are the preferred choices for treating symptomatic episodes of AF. Class III antiarrhythmic drugs are usually insufficient for AF control (sotalol) or relatively contraindicated in a young population due to side effects (amiodarone). Class Ia drugs such as disopyramide and Class Ic drugs such as flecainide and propafenone are the preferred options for athletes with exercise-induced AF who do not have structural heart disease. Using the “pill-in-the-pocket” strategy has a high rate of compliance by patients, a low rate of adverse events, and a significant reduction in emergency room visits and hospital admissions [68]. Small studies with disopyramide in patients’ post-cardioversion have shown that this drug can maintain sinus rhythm in 67% of patients at six months and 54% at one year due to its anticholinergic effects [69]. When compared, propafenone is better tolerated than disopyramide, which may cause heart failure [70].

Class I should not be used in monotherapy since these drugs may tend to develop atrial flutter, which, in the absence of adequate rate control, may lead to 1:1 atrioventricular conduction, very high ventricular rates, and profound intraventricular conduction slowing with hemodynamic compromise (Class III, Level of Evidence C) [48,70,71]. Flecainide or propafenone can be used as a “pill-in-the-pocket” strategy for EA with paroxysmal AF after careful initiation and assessment of drug effects. However, participation in intensive sports is not recommended until two half-lives of the antiarrhythmic drug have elapsed (up to 2 days) due to their pro-arrhythmic potential caused by adrenergic hyperactivity during exercise (Class III, Level of Evidence C) [71]. Combining flecainide with calcium channel blockers such as Diltiazem or Verapamil may also help prevent secondary atrial flutter [71].

### 5.3. Spontaneous Cardioversion

Spontaneous conversion to sinus rhythm in patients with symptomatic atrial fibrillation (AF) is common, especially in cases of recent-onset or paroxysmal AF, where episodes self-terminate within 48 h [72]. This likelihood is higher in younger patients and those without underlying heart disease as EA. The potential for spontaneous conversion informs clinical decisions, as a “wait-and-see” approach may be safe for stable patients without severe symptoms, allowing time for natural rhythm restoration before considering active cardioversion. The proposed scores for spontaneous conversion in sinus rhythm may help physicians tailor AF management in an effective and timely manner. If conversion does not occur within 24–48 h, however, medical intervention may be necessary to restore normal heart rhythm [73].

### 5.4. Direct Cardioversion

Electrical cardioversion is the preferred treatment for athletes who experience disabling symptoms or hemodynamic instability, particularly in the absence of structural heart disease. After cardioversion, a follow-up strategy must be developed to guide further therapy. Anticoagulation management for endurance athletes follows the same guidelines as those for the general population [5,6].

### 5.5. Nonpharmacological Treatment

The data on radiofrequency (RF) catheter ablation of AF in endurance athletes are limited [5,74] but support the effectiveness of this treatment approach. Long-term follow-up is necessary to confirm the sustainability of these benefits over the years.

An initial study of 20 athletes (average age 44.4 ± 13.0 years) reported that all participants were free from AF and no longer required antiarrhythmic therapy 36.1 ± 12.7 months after pulmonary vein isolation. The athletes showed a significant increase in exercise capacity (from 183 ± 32 to 218 ± 20 W, *p* < 0.02). Furthermore, after six months, all athletes were cleared for competitive sports and experienced substantial improvements in several quality-of-life indicators [75].

Calvo et al. compared the effectiveness of pulmonary vein isolation (PVI) in two groups: EA with lone AF and other patients with AF. There were no significant differences in procedural effectiveness between the two groups. The study found that LA diameter and long-standing AF were the only independent predictors of arrhythmia recurrence [76]. Furlanello et al. demonstrated a 90% success rate after an average of two RF ablation procedures in a series of 20 athletes, with no major complications. The study also aimed to help veteran athletes (average age 44 ± 13 years) resume competitive activities, which all participants successfully achieved [77]. Studies have also shown similar proportions of arrhythmia-free patients when comparing endurance and non-endurance athletes three years after repeated RF ablation procedures.

An early invasive approach using the latest ablation techniques of AF is recommended in exercising individuals with recurrent symptomatic AF or in athletes who do not want drug therapy due to its impact on athletic performance (Class I, Level of Evidence B) [48,76,78,79]. Substantial groups of athletes may quickly develop an intolerance to medical therapy, leading to nonpharmacologic treatment. Additionally, cavotricuspid isthmus ablation may be considered in EA with known atrial flutter motivated to engage in intensive exercise to prevent 1:1 atrial flutter (Class IIa, Level of recommendation C) [48].

There is no substantial evidence about the safe dose of sports after RF ablation. Physical activity may be resumed if no arrhythmia recurrences are documented within one month of a procedure. Questions arise regarding the continuation of sports after successful PVI, which might progress the disease and lead to the recurrence of AF in the future.

### 5.6. Anticoagulation

The initiation of anticoagulant therapy in athletes depends on their stroke risk factors, which are assessed using the CHA2DS2-VASc score (congestive heart failure, hypertension, age > 75 years, diabetes mellitus, prior stroke or transient ischemic attack or thromboembolism, vascular disease, age 65 to 74 years, and sex category), based on the ESC 2024 AF Guidelines CHA2DS2-VA score and the risk of bleeding, evaluated through scores such as HAS-BLED (hypertension, abnormal renal and liver function, stroke, bleeding, labile international normalized ratio, elderly, drugs or alcohol use) or ATRIA (Anticoagulation and Risk Factors in Atrial Fibrillation) [5,6,19]. Many endurance athletes may lack established stroke risk factors and thus have low scores. If anticoagulation is deemed necessary, it is recommended to assess bleeding risk within the context of the specific sport [80].

Sports with direct bodily contact or prone to trauma are not recommended in exercising individuals with AF who are anticoagulated [48]. Sports such as rugby, cycling, or motorsports can cause a significant risk of harm and bleeding [80].

## 6. Conclusions

The cardiovascular benefits of exercise are well-established, and athletes are often viewed as symbols of health in contemporary culture. However, the occurrence of AF in endurance athletes raises concerns that even a healthy lifestyle can have harmful effects when taken to extremes. Significant gaps exist in understanding the epidemiology, mechanisms, and treatment of AF in EA. Potential mechanisms may include atrial enlargement, fibrosis, and increased vagal tone. Evidence suggests that fibrosis related to endurance training may be reversible in its early stages. Treatment decisions for this population must be based on the best available data and clinical judgment, as well as careful clinical judgment. Treatment for AF in this group may differ from that for sedentary individuals or the general population, but it typically includes strategies to manage both the rhythm disturbance and the athlete’s training intensity. Proper reduction of exercise intensity when AF is diagnosed is the biggest challenge for clinicians, no matter whether drug therapy or RF ablation is the treatment strategy. Given the unique identity and lifestyle considerations of EA, treatment choice should respect their preferences, balancing the need for medical intervention with a desire to continue physical activity. Supporting athletes in maintaining moderate exercise can have a positive impact on their mental health and overall quality of life, as exercise remains a significant part of their identity and well-being.

Further studies are essential to deepen the understanding of the pathophysiological mechanisms and to develop tailored management algorithms for AF in EA.

## Figures and Tables

**Table 2:** Imaging modalities for assessment of left atria.

Imaging Modality	Parameters	Strengths	Limitations
Echocardiography (TTE)	E/A Ratio: The E/A ratio evaluates left ventricular diastolic function, which affects left atrial (LA) pressure and size. A high E/A ratio indicates reduced compliance and diastolic dysfunction. Left Atrial Function Index (LAFI): LAFI combines atrial volume and function to assess LA reservoir performance. It is useful for distinguishing between adaptive and maladaptive atrial enlargement, particularly in conditions like atrial fibrillation. Left Atrial Volume Index (LAVI): LAVI measures LA size adjusted for body surface area, providing insight into LA remodeling and an established predictor for cardiovascular events. Strain Imaging: LA strain, typically assessed using speckle-tracking echocardiography, evaluates LA reservoir, conduit, and contractile function. Decreased strain indicates reduced LA compliance and function, often seen in atrial fibrillation.	Non-invasive and readily accessible: Echo is widely available, inexpensive, and can be performed at the bedside. Real-time assessment: Provides dynamic information on atrial and ventricular function. Functional metrics: Allows assessment of diastolic function and strain imaging, which is highly informative for tracking LA dysfunction in the athlete population.	Operator-dependent: Image quality and interpretation can vary significantly based on the operator’s skill. Limited spatial resolution: May not provide as detailed anatomical information on LA walls as MRI or CT. Limited view of fibrosis: Cannot reliably detect LA fibrosis or scar tissue, which are better assessed with MRI.
Magnetic Resonance Imaging (MRI)	MRI offers detailed anatomical and functional assessment of the LA, providing valuable information on LA size, morphology, and fibrosis. LA fibrosis can be assessed through late gadolinium enhancement (LGE) MRI, which detects scarring in the atrial wall, relevant for atrial fibrillation management and risk stratification.	High spatial resolution: Offers superior image quality and detailed visualization of the LA structure. Fibrosis detection: LGE can detect fibrosis and scarring, aiding in risk stratification for atrial fibrillation recurrence. Quantitative analysis: Provides precise measurements of LA volume and function.	Cost and availability: MRI is more expensive and less accessible than echo. Longer examination time: The scan takes significantly longer, which may be problematic for people with claustrophobia. Gadolinium use: Contrast agents may be contraindicated.
Computed Tomography (CT)	CT imaging provides an accurate assessment of LA size and anatomy. It is commonly used in planning atrial fibrillation ablation procedures, as it can precisely map the LA and pulmonary vein anatomy.	High-resolution anatomical detail: Excellent for visualizing LA anatomy, especially in pre-ablation planning. Quick scan time: CT scans are rapid, making them convenient for patients struggling with longer imaging sessions.	Radiation exposure: CT involves radiation, which is a concern for repeated assessments. Limited functional assessment: CT does not assess LA function, strain, or fibrosis as effectively as MRI or echo. Contrast requirements: Iodinated contrast agents, required for enhanced imaging, are not suitable for all patients.

## Data Availability

No new data were created or analyzed in this study. Data sharing is not applicable to this article.
